# Microstructure Evolution in ZrC*_x_* with Different Stoichiometries Irradiated by Four MeV Au Ions

**DOI:** 10.3390/ma12223768

**Published:** 2019-11-16

**Authors:** Boxin Wei, Dong Wang, Yujin Wang, Haibin Zhang

**Affiliations:** 1 School of Materials Science and Engineering, Harbin University of Science and Technology, Harbin 150040, China; weiboxin@hrbust.edu.cn; 2 Institute for Advanced Ceramics, School of Materials Science and Engineering, Harbin Institute of Technology, Harbin 150001, China; 3 Key Laboratory of Advanced Structural-Functional Integration Materials & Green Manufacturing Technology, Harbin Institute of Technology, Harbin 150001, China; 4 Key Laboratory of Advanced Manufacturing and Intelligent Technology, Ministry of Education, Harbin University of Science and Technology, Harbin 150040, China; 5 Heilongjiang Provincial Key Laboratory of Light Metal Materials Modification and Green Forming Technology, Harbin University of Science and Technology, Harbin 150040, China; 6 Innovation Research Team for Advanced Ceramics, Institute of Nuclear Physics and Chemistry, China Academy of Engineering Physics, Mianyang 621900, China

**Keywords:** zirconium carbide, carbon vacancy, ion irradiation, defects

## Abstract

ZrC*_x_* ceramics with different stoichiometries were irradiated under a four MeV Au ion beam in doses of 2 × 10^16^ ions/cm^2^ at room temperature, corresponding to ~130 dpa. Grazing incidence, X-ray diffraction and transmission electron microscopy were performed to study the radiation damage and microstructure evolution in ZrC*_x_* ceramics. With the decrease in C/Zr ratio, the expansion of ZrC*_x_* lattice became smaller after irradiation. Some long dislocation lines formed at the near-surface, while, in the area with the greatest damage (depth of ~400 nm), large amounts of dislocation loops formed in ZrC, ZrC_0.9_ and ZrC_0.8_. With the increase in carbon vacancy concentration, the size of the dislocation loops gradually decreased. Few dislocation loops were found in ZrC_0.7_ after irradiation, and only black-dot defects were found in the area with the greatest damage. For the non-stoichiometric ZrC*_x_*, with the increase of the intrinsic vacancies, the number of C interstitials caused by irradiation decreased, and the recombination barrier of C Frenkel pairs reduced. The above factors will reduce the total number of C interstitials after cascade cooling, suppressing the formation and growth of dislocation loops, which is significant for the enhancement of the tolerance of radiation damage.

## 1. Introduction

Due to the combination of the high temperature, high neutron irradiation dose and extremely corrosive environment of Generation IV nuclear reactor systems, the development of advanced nuclear materials with good radiation resistance, corrosion resistance and high thermostability is urgent [1]. Silicon carbide (SiC) is considered as a potential material for nuclear materials due to its extraordinary resistance to irradiation [2]. However, there are some limitations for the use of SiC in Generation IV nuclear reactor systems. SiC could transform from a *β*-SiC to *α*-SiC under the accident conditions, which could result in failure of the materials and the release of fission products [3]. In addition, the SiC is susceptible to attack by palladium, which can potentially compromise the retention of fission products [4,5].

Zirconium carbide (ZrC) has been considered as a Tri-structural iso-tropic (TRISO)-coating fuel particle, fuel cladding or an inert matrix material, due to its high melting point, high thermal conductivity, low neutron absorption cross-section and excellent resistance to attack by fission products [6,7,8]. As a member of the family of transition metal carbides, ZrC has an NaCl crystal structure which is stable over a relatively wide compositional range of 0.6 to 1.0 [9]. The properties of ZrC*_x_* ceramics are generally sensitive to the C/Zr atom ratio [10,11]. As is known, stoichiometry is a critical factor for the properties of ZrC*_x_* [12]. Thus, the carbon vacancies will likely be an important factor affecting the irradiation behavior of ZrC*_x_*.

Many studies on the microstructure evolution of stoichiometric ZrC under irradiation have been carried out. In situ irradiations of ZrC_1.01_ were performed by Gan et al. [13] using Kr irons to 10 and 30 displacements per atom (dpa) at room temperature, and 10 and 70 dpa at 800 °C, and observing the formation of a high density of black-dot defects at room temperature and dislocation segments at 800 °C. Yang et al. [14] conducted ion irradiations of ZrC up to 0.7 and 1.5 dpa at 800 °C using 2.6 MeV protons and found the formation of Frank loops. Additionally, Gosset et al. [15] found a high density of defects of a certain size, which evolved into a dislocation network when the dose was increased by using four MeV Au ions at room temperature. Single crystals ZrC were irradiated with 1.2 MeV Au ions for various doses in the range 2 × 10^14^–3 × 10^16^ ions/cm^2^ by Pellegrino et al. [16] at room temperature, and dislocation loops for doses above 10^15^ ions/cm^2^ were observed. Agarwal et al. [17] performed three MeV He^+^ ion irradiations up to 5 × 10^20^ ions/m^2^ and high-temperature annealing (1000–1600 °C) and found that, underneath blister caps, the microstructure of ZrC evolved into ultra-fine nano-scale grains consisting of numerous nano-cracks at 1500 °C. Snead et al. [18] conducted fast neutron irradiations of ZrC for the fluences of 0.8–9.38 × 10^25^ neutrons/m^2^ at temperatures ranging from 635 to 1496 °C, using the High Flux Isotope Reactor. It was found that the dislocation loops transitioned from Frank to prismatic loops in ZrC at higher temperatures. Some investigations on the effects of stoichiometry on the irradiation response in ZrC*_x_* have been carried out using proton irradiations to 1–3 dpa at 800 °C [19] and 2 dpa 1125 °C [20]. In our previous study [21], we found that the superstructure modulation of the ordered carbon vacancies for Zr_2_C in ZrC_0.6_ was destroyed under Au ion irradiation. However, this is limited for the understanding of the effects of stoichiometry of ZrC on radiation damage, and no systematic investigation has been performed on the effect of stoichiometry on the radiation damage in ZrC*_x_*.

In the present studies, the effect of stoichiometry on damage resistance and microstructure evolution of ZrC*_x_* under irradiation were investigated using a four MeV Au ion beam in fluences of 2 × 10^16^ ions/cm^2^ at room temperature. Grazing incidence X-ray diffraction (GIXRD) and transmission electron microscopy (TEM) were performed to study the radiation damage and microstructure evolution. The influence mechanism of C vacancies on irradiation defects was also discussed. The fundamental understanding of the microstructure evolution over a range of stoichiometry will provide a baseline for the applications of ZrC*_x_* in Generation IV nuclear reactor systems.

## 2. Materials and Methods

### 2.1. Materials

Non-stoichiometric ZrC*_x_* ceramics were prepared by two-step reactive hot pressing in our previous study [11]. Commercially available powders of ZrC (purity >99.5 wt.%, particle size 1–5 μm, Changsha Weihui Materials Company, Changsha, China) and ZrH_2_ (purity >99.6 wt.%, particle size 2–10 μm, Jinzhou Haixin Metal Materials Company, Jinzhou, China) were chosen as starting powders. Mixed powders with appropriate ratios were ball-milled in ethanol by ZrO_2_ milling balls with a speed of 300 rpm for 24 h. Then, a rotary evaporator was used to dry the slurry, and the dried powders were sieved by a 200-mesh screen. The ZrC*_x_* ceramics were sintered by a two-step reactive sintering method, including a low-temperature reaction (1300 °C for 30 min) for the decomposition of ZrH_2_ and outgassing of H_2_ and then a high-temperature densification by hot pressing. The following reactions occur at a relatively low temperature:

ZrH_2_ → Zr + H_2_↑ (1)

(1 − *x*)Zr + *x*ZrC → ZrC*_x_* (*x* = 0.7~1) (2)

The reactions of Equations (1) and (2) will form ZrC*_x_* phase with a composition parameter *x* of 0.7 to 1.0 after completion. The basic properties of ZrC*_x_* ceramics used in this study are shown in Table 1.

### 2.2. Au Ions Irradiation

Specimens of dimensions of 3 × 4 × 5 mm^3^ were cut from the ceramics. The surfaces of specimens were polished before irradiation. The irradiations of ZrC*_x_* were performed on the 5SDH-2 accelerator (Peking University, Beijing, China) using four MeV gold ions of a fluence of 2 × 10^16^ ions/cm^2^ at room temperature, with the beam current held below 1 μm·cm^–2^ in order to avoid significant bulk heating. The chamber was maintained in vacuum with a pressure <10^–3^ Pa during irradiation, and the ion beam direction was set perpendicular to the irradiated surface. The number of dpa of the ceramics after irradiation along the depth was calculated with the software of the stopping and range of ions in matter version 2013 (SRIM-2013) in full cascade mode, using displacement energies of 37 and 16 eV for Zr and C [22], respectively. The input parameters for SRIM simulation are shown in Table 1.

### 2.3. Characterization

Grazing incidence X-ray diffraction (GIXRD, Empyrean, PANaytical Corp., Almelo, The Netherlands) using CuK*_α_* radiation was chosen to analyze the changes of crystal structure during irradiation. The diffractometer was operated with a glancing angle of 0.7° and a scanning speed of 1°/min.

Focused ion beam (FIB) lift-out transmission electron microscopy (TEM) samples were prepared using HELIOS NanoLab 600i (FEI, Hillsboro, OR, USA). Firstly, an electron beam with 30 keV assisted deposition of platinum was applied at a position of interest with a thickness of 0.5 μm, followed by an ion beam-assisted deposition of platinum with a thickness of 1–2 μm, aiming to reduce the gallium ion contamination of the top face of the sample. Then, a sheet with a thickness of 0.5 μm was cut out with a beam current of 20 nA. When the desired thickness was cut out, the sheet was welded to a micro-mechanical hand, and then thinned to 300 nm with an ion beam of 30 kV and 100 pA. Next, an ion beam of 10 kV and 50 pA was used to obtain a thickness of 150 nm, and finally a thickness of ~80 nm was cut by an ion beam of 5 kV and 10 pA. TEM (FEI Talos F200x, Eindhoven, Netherlands) was used for a more detailed analysis of the microstructure.

## 3. Results

### 3.1. Irradiation Damage Simulation

**Figure 1:**
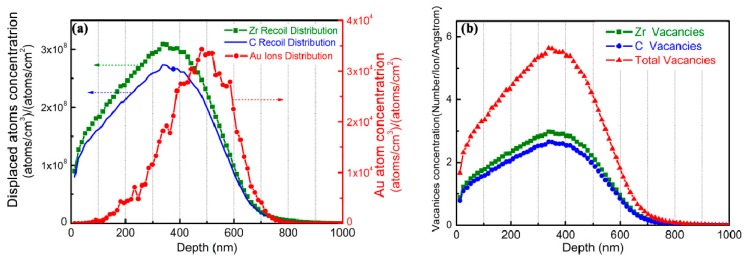
Distribution of (**a**) displaced Zr and C atoms and (**b**) Zr vacancies and C vacancies in ZrC irradiated by a beam of 10,000 four MeV Au ions calculated with SRIM.

The SRIM estimation of damage of ZrC is shown in Figure 1, which shows the distribution of Au ions, displacement atoms and vacancies in irradiated ZrC. It can be seen that the numbers of displaced Zr atoms and C atoms are much higher than that of Au ions. These displacement atoms consist of Zr atoms and C atoms that vacated their lattice positions. It is worth noting that the peaks of displaced Zr atoms and C atoms are shallower than the concentration peaks of Au ions. This is because, near the end of Au ion track, the ion energy is insufficient to generate a large amount of displacement damage. Figure 1b shows the distribution of Zr vacancies and C vacancies along the depth from the surface of ZrC, caused by Au ion irradiation. The total number of Zr vacancies is slightly higher than that of C vacancies, and the concentrations of vacancies reach their highest value in the depth range of 300–400 nm, where they withstand the most serious damage.

**Figure 2:**
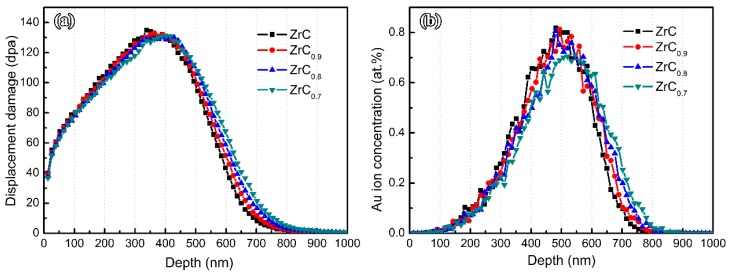
SRIM prediction for (**a**) damage production and (**b**) implanted Au distributions for ZrC*_x_* with different stoichiometries, calculated with SRIM under irradiation of a fluence of 2 × 10^16^ ions/cm^2^ by a four MeV Au ion beam.

**Figure 3:**
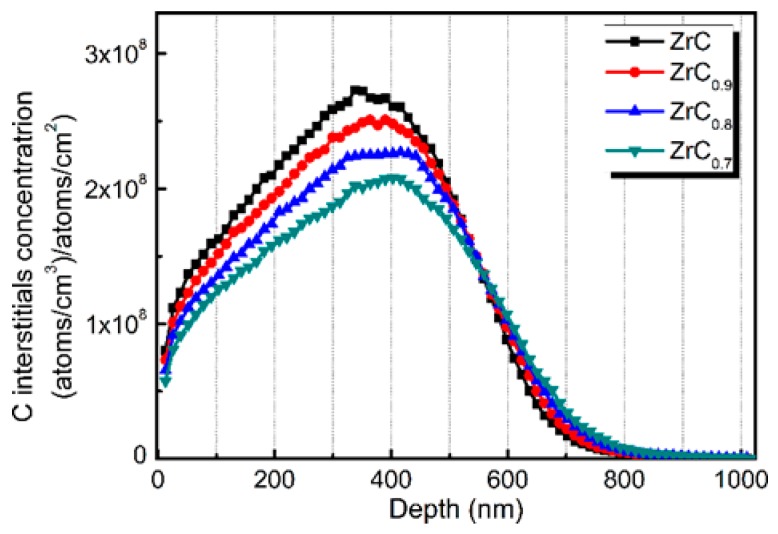
C interstitials concentration vs. depth in ZrC*_x_* irradiated by a beam of 10,000 four MeV Au ions calculated with SRIM.

The SRIM estimation for damage production and implanted Au distributions for ZrC*_x_* with different stoichiometries are shown in Figure 2. The distribution of Au ions along the depth from the surface of ZrC*_x_* agrees with the Gaussian distribution, and the incident depth of Au ions was less than 900 nm. Comparing the depth of radiation damage for ZrC*_x_* with different stoichiometries, the depth of radiation damage gradually increases as the C/Zr ratio decreases. The damaged depth in irradiated ZrC was about 850 nm, while that in irradiated ZrC_0.7_ was slightly deeper, reaching 900 nm. This is mainly because the atomic density of ZrC*_x_* gradually decreases as the C/Zr ratio decreases: from 7.69 × 10^22^ atoms/cm^3^ of ZrC to 6.48 × 10^22^ atoms/cm^3^ of ZrC_0.7_. The steric hindrance and energy loss of Au ions could be reduced by a lower atomic density. Additionally, the highest value of radiation damage in ZrC was ~135 dpa, while that in ZrC_0.7_ was slightly lower, at 130 dpa. This is because the simulation of the irradiation process is based on the ratio of the probability of Au ions colliding with Zr atoms and C atoms. The displacement energy of the C atoms given by the SRIM program was lower than that for Zr atoms, and the ratio in the ZrC_0.7_ sample is 10:7. Thus, it is predicted by the simulation that ZrC_0.7_ had a lower dpa, as shown in Figure 2a. C interstitials concentration along the depth in ZrC*_x,_* irradiated by a beam of 10,000 four MeV Au ions, is shown in Figure 3. It can be seen that, as the intrinsic C vacancies increase, the concentration of C interstitials generated by irradiation decreases. Overall, as the C/Zr ratio decreases, the probability of the collision of Au ions with Zr atoms in ZrC*_x_* becomes higher, which slightly decreases the highest radiation damage value.

### 3.2. Lattice Parameter Changes

**Figure 4:**
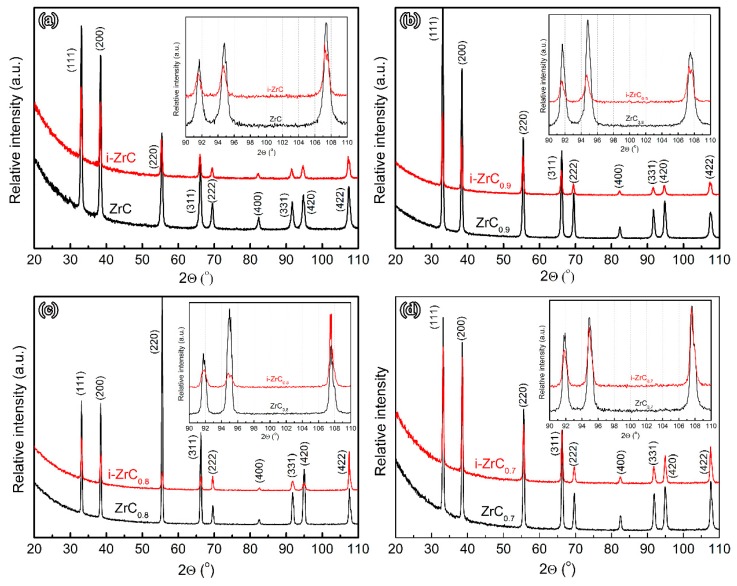
Grazing incidence X-ray diffraction (GIXRD) of ZrC*_x_* with different stoichiometries before and after irradiation. (**a**) ZrC; (**b**) ZrC_0.9_; (**c**) ZrC_0.8_; (**d**) ZrC_0.7_.

The damage of ZrC*_x_* ceramics under Au ions is mainly due to the large number of displacement atoms and vacancies generated by irradiation. Although most of the displacement atoms can return to inherent lattice vacancies, unreacted displacement atoms and vacancies will cause corresponding changes to the crystal structure and lattice parameter. Therefore, GIXRD can be used to evaluate the degree of radiation damage of ZrC*_x_* by calculating the change in lattice parameter before and after irradiation. Figure 4 shows GIXRD patterns of ZrC*_x_* with different stoichiometries before and after irradiation. It can be seen that no new peak appears in the GIXRD pattern of ZrC*_x_* after irradiation, indicating that no decomposition and amorphous transformation of ZrC*_x_* occurred during irradiation.

It can be found that the peaks of ZrC*_x_* are broadened with weakened intensities after irradiation. The weakening of the peak intensity indicates that the defects caused by Au ion irradiation cause some damage to the crystal structure. The broadening of the diffraction peak may be caused by the micro-strain and point defects and dislocations in the ZrC*_x_* lattice caused by irradiation [14,23,24,25,26].

In addition, the position of the diffraction peak of ZrC*_x_* after irradiation also changed. In the magnified patterns at high angles very small shifts of peaks from high angles to low angles in GIXRD patterns were observed. The offsets become smaller as the C/Zr ratio decreases. The decrease in the 2θ angle indicates an increase in the corresponding d-spacing [27]. Hence, the ZrC*_x_* lattice was expanded after irradiation. The lattice parameters of ZrC*_x_* before and after irradiation were calculated from the diffraction patterns and are shown in Table 2. The lattice parameter of ZrC increased from 4.6815 ± 0.0013 Å to 4.6870 ± 0.0007 Å, while that of ZrC_0.7_ increased only from 4.6776 ± 0.0011 Å to 4.6785 ± 0.0024 Å. With the decrease in the C/Zr ratio, the lattice expansion of ZrC*_x_* after irradiation becomes smaller, the lattice expansion of ZrC is 0.117%, and the lattice swelling of ZrC_0.7_ is only 0.019%. According to the previous SRIM simulation results, a large number of interstitial atoms were generated during Au ion irradiation. Defects formed by these interstitials cause lattice distortion, causing the lattice swelling of ZrC*_x_*. With the increase in the concentration of intrinsic C vacancies in ZrC*_x_*, a large number of intrinsic C vacancies can interact with the interstitials generated by irradiation, meaning that the interstitials return to the intrinsic C vacancies, inhibiting the formation of irradiation defects and reducing the lattice distortion, which can restrain the lattice expansion.

### 3.3. Microstructure Changes

#### 3.3.1. ZrC

**Figure 5:**
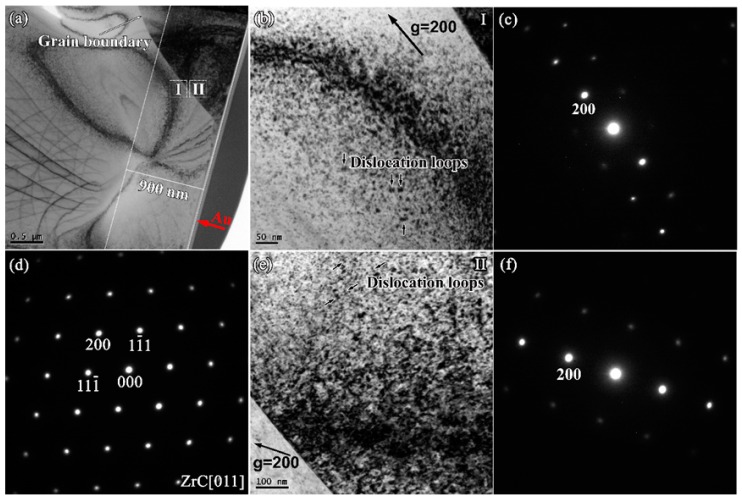
(**a**) Cross-section bright field (BF)-TEM images of ZrC after irradiation; (**b**) BF image of irradiated ZrC at region I taken from (**c**) a two-beam condition diffraction pattern with g = 200 near zone axis [011]; (**d**) Selected area electron diffraction (SAED) patterns obtained from the zone axis [011] of the damaged area; (**e**) BF image of irradiated ZrC at region II taken from (**f**) a two-beam condition diffraction pattern with g = 200 near the zone axis [001].

Figure 5a shows a cross-section bright field (BF) TEM image of ZrC after irradiation, in which a red arrow highlights the implant direction and white lines show the surface and depth of damage. It can be observed that the depth of the distinct damage layer is 900 nm, which matches with the results of SRIM simulations. Figure 5d shows the selected area electron diffraction (SAED) pattern obtained from the zone axis [011] of the damaged area. The diffraction pattern indicates that the ZrC crystal structure is intact after irradiation and no amorphization occurs. A two-beam condition was chosen in TEM analysis because the diffraction vector and the defect image can be easily correlated to determine the defect type under the condition. From the BF image of the irradiated ZrC (Figure 5b) taken from a two-beam condition with g = 200 near the zone axis [011], it can be observed that the dislocation loops were generated after irradiation. Dislocation loops with a high density were also found in the BF image taken from the two-beam condition with g = 200 near the zone axis [001], as shown in Figure 5e, indicating that the dislocation loops with high-density form in the area with the greatest damage (~400 nm from the surface) after Au ion irradiation, and the average size of the dislocation loops is 18.1 ± 5.6 nm.

**Figure 6:**
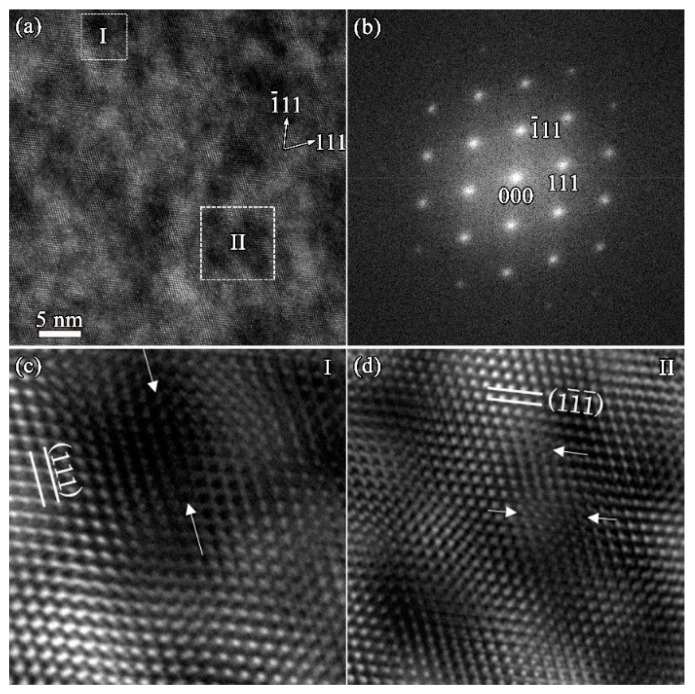
(**a**) High-resolution (HR)-TEM images of the damaged area interior obtained from the zone axis [011] in irradiated ZrC; (**b**)Fourier transformation of (**a**); (**c**) Fourier-filtered (111) diffraction lattice images; (**d**) Fourier-filtered (1$\overset{-}{1}$ $\overset{-}{1}$) diffraction lattice images.

Further, the microstructure of the defect generated by irradiation in the area with the greatest damage is analyzed. Figure 6a shows the high-resolution (HR) TEM images of the damaged area’s interior obtained from the zone axis [011] in irradiated ZrC. The lattice fringes were found to be distorted, which was attributed to the lattice stress caused by the irradiation damage, indicating that there is not only a larger lattice constant but also a distortion of its lattice in ZrC after irradiation. Figure 6b shows the corresponding Fourier transform pattern. According to the Fourier deconvolution, Fourier-filtered (111) and (1$\overset{-}{1}$$\overset{-}{1}$) diffraction lattice images were obtained, respectively, as shown in Figure 6c and d. Dislocations can be observed in the high-resolution transmission electron microscopy (HRTEM) image after deconvolution along (111) and (1$\overset{-}{1}$$\overset{-}{1}$), and the interstitials can cluster along the (1$\overset{-}{1}$$\overset{-}{1}$) crystal plane to form a dislocation loop. Therefore, a large number of dislocation/dislocation loop defects were generated in ZrC under four MeV gold ion irradiation (2 × 10^16^ ions/cm^2^).

#### 3.3.2. ZrC_0.9_

**Figure 7:**
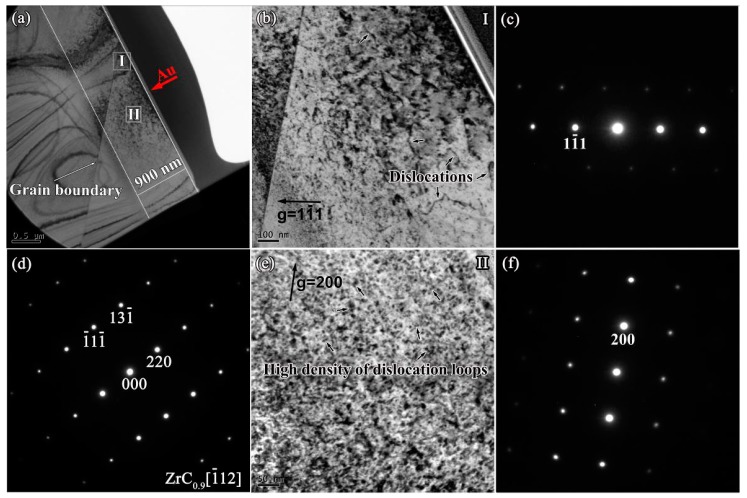
(**a**) Cross-section BF-TEM images of ZrC_0.9_ after irradiation; (**b**) BF image of irradiated ZrC_0.9_ at Region I taken from (**c**) a two-beam condition diffraction pattern with g = 1$\overset{-}{1}$1 near the zone axis [011]; (**d**) SAED patterns obtained from the zone axis [$\overset{-}{1}$12] of the damaged area; (**e**) BF image of irradiated ZrC_0.9_ at region II taken from (**f**) two-beam condition diffraction pattern with g = 200 near zone axis [001].

In order to investigate the effect of C vacancies on the defects of ZrC*_x_* after irradiation, TEM analysis was carried out on the cross sections of ZrC_0.9_, ZrC_0.8_ and ZrC_0.7_ along the depth directions after irradiation. Figure 7a and d show the cross-section BF-TEM images and SAED patterns in the damaged area of ZrC_0.9_ after irradiation. The depth of the damaged region of ZrC_0.9_ is close to that of ZrC, which is about 900 nm, and the crystal structure of ZrC_0.9_ after irradiation is also intact. However, the microstructure of defects in the near-surface area is different from that in the damaged area after irradiation. According to the BF-TEM images, under a two-beam condition within the near surface, as shown in Figure 7b, long dislocation lines were observed, and the amount of dislocation was relatively low. Figure 7e shows the defect morphology under the two-beam condition within the interior of the damaged layer, which is similar to the morphology of the defect in ZrC. More dislocation loops appear in the area with the greatest damage, and the average size of the loops is 11.3 ± 2.8 nm.

#### 3.3.3. ZrC_0.8_

**Figure 8:**
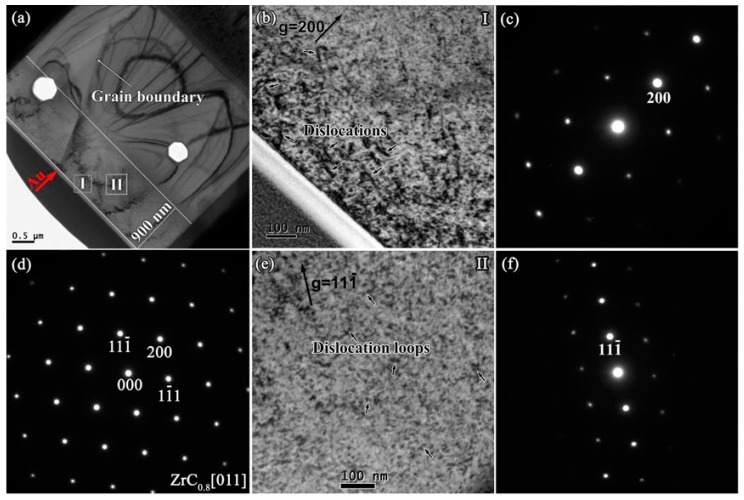
(**a**) Cross-section BF-TEM images of ZrC_0.8_ after irradiation; (**b**) BF image of irradiated ZrC0.8 at Region I taken from (**c**) a two-beam condition diffraction pattern with g = 200 near the zone axis [011]; (**d**) SAED patterns obtained from the zone axis [011] of the damaged area; (**e**) BF image of irradiated ZrC_0.8_ at region II taken from (**f**) a two-beam condition diffraction pattern with g = 11$\overset{-}{1}$ near the zone axis [011].

Figure 8a,d show the cross-section BF-TEM images and SAED patterns in the damaged area of ZrC_0.8_ after irradiation. Similar to ZrC and ZrC_0.9_, the crystal structure of ZrC_0.8_ is maintained after irradiation, and no amorphization occurs. The microstructure of defects at the near-surface area and the damage area after irradiation are similar to those in ZrC_0.9_ The defects in the near-surface area are mainly composed of some long dislocation lines (Figure 8b), while a large amount of dislocation loops were observed within the interior of the damaged layer (Figure 8e) with an average size of 11.1 ± 2.6 nm, which is smaller than those in ZrC and ZrC_0.9_. This is because the existence of the C vacancies can absorb the C interstitial generated by the irradiation, effectively suppressing the nucleation and growth of the dislocation loop, and, as the concentration of C vacancies increases, the suppression effect increases, resulting in a slight decrease in the size of the dislocation loops [23]. In addition, some clear-faceted cavities were seen in Figure 8a. We are not sure of the reason for the clear-faceted cavities, it may be noted that impurities could result in the cavities during the preparation of FIB lift-out TEM samples.

The defect structures in ZrC, ZrC_0.9_ and ZrC_0.8_ are similar after irradiation. The NaCl type crystal structure of ZrC is maintained after irradiation, and some long dislocation lines are formed at the near surface. In the area with the greatest damage (depth of ~400 nm), large amounts of dislocation loops formed, and, as the concentration of C vacancy increased, the size of the dislocation loops gradually decreased from 18.1 ± 5.6 nm to 11.1 ± 2.6 nm.

#### 3.3.4. ZrC_0.7_

**Figure 9:**
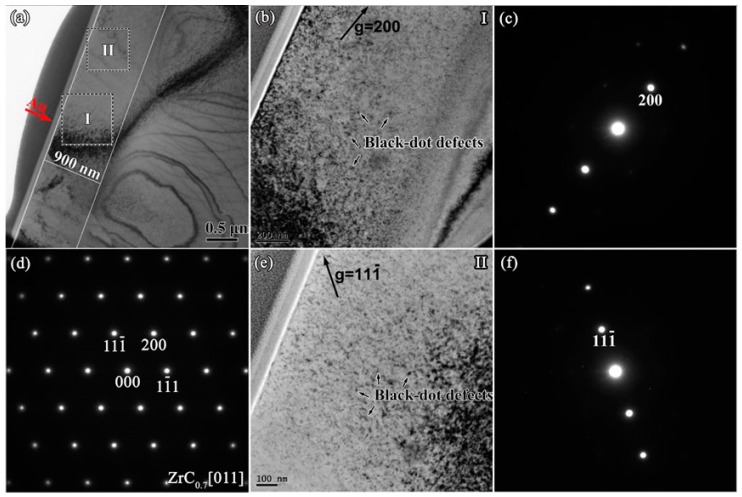
(**a**) Cross-section BF-TEM images of ZrC_0.7_ after irradiation; (**b**) BF image of irradiated ZrC_0.7_ at Region I, taken from (**c**) a two-beam condition diffraction pattern with g = 200 near the zone axis [011]; (**d**) SAED patterns obtained from the zone axis [011] of the damaged area; (**e**) BF image of irradiated ZrC_0.7_ at region II taken from (**f**) a two-beam condition diffraction pattern with g = 11$\overset{-}{1}$ near the zone axis [011].

However, for ZrC_0.7_, the defect structure after irradiation is different from those of ZrC, ZrC_0.9_ and ZrC_0.8_. It can be seen from the change of lattice parameter in Section 3.1 that the lattice swelling of ZrC_0.7_ is only 0.019%, which is much smaller than those of ZrC, ZrC_0.9_ and ZrC_0.8_, indicating that ZrC_0.7_ has a better radiation resistance. According to Figure 9a,d, after irradiation, the crystal structure of ZrC_0.7_ was intact and no amorphization occurred. Few dislocation loops were found in the BF images with g = 200 and g = 11$\overset{-}{1}$ in the damage region, as shown in Figure 9b,e, and only black-dot defects were found in in the area with the greatest damage. This is because there are more intrinsic C atom vacancies in ZrC_0.7_. Most of the interstitials generated by Au ion irradiation will combine with vacancies after cascade cooling, and the presence of intrinsic C vacancies will increase interstitials. The probability of combining with vacancies reduces the presence of interstitials and inhibits the formation of dislocation loops. Point defects, such as a large number of vacancies and a small number of interstitials, appear as "black-dot" defects in the TEM images.

In summary, there are similar microstructures of defects in ZrC, ZrC_0.9_ and ZrC_0.8_ after irradiation. The NaCl type crystal structure of maintained after irradiation, and some dislocations formed at the near surface. In the area with the greatest damage (depth of ~400 nm), large amounts of dislocation loops formed, and as the C vacancy concentration increased, the size of the dislocation loops gradually decreased from 18.1 ± 5.6 nm to 11.1 ± 2.6 nm. No obvious dislocation loop was found in ZrC_0.7_ after irradiation. In the area with the greatest damage, only "black-dot" defects were found. The existence of a large number of intrinsic C vacancies increases the probability of the combination of interstitials and vacancies generated by irradiation, resulting in a reduction in the existence of interstitials and the inhibition of the formation of dislocation loops.

## 4. Discussion

Ion beam irradiation is commonly used to simulate radiation effects in materials used in advanced nuclear energy systems [28]. The ZrC*_x_* lattice was expanded after Au ion irradiation, and this was also found in ZrC irradiated in proton irradiation (2.6 MeV) [14]. With the decrease in C/Zr ratio, the lattice expansion of ZrC*_x_* after irradiation decreases. The effects of stoichiometric variation on swelling of ZrC lattice during irradiation are reported. The defect structures in ZrC, ZrC_0.9_ and ZrC_0.8_ are similar after irradiation. The NaCl type crystal structure of ZrC was maintained after irradiation. No irradiation-induced voids were observed, which is consistent with the results of Yang et al. [14], Gan et al. [13,29], and Gosset et al. [15]. 

Generally, ion irradiation affects the microstructure and properties of materials according to the interstitials and vacancies generated by the irradiation. First, different kinds of defects are formed by the interstitials and vacancies clustering under certain conditions; further, the defects have an impact on the properties of materials. In order to investigate the influence mechanism of C vacancies on the irradiation damage behavior of ZrC*_x_* ceramics, the following questions should be clarified: (1) what are the kind, quantity and distribution of interstitials and vacancies generated by Au ions in irradiated ZrC? (2) How do these interstitials and vacancies move after cascade cooling? How many interstitials and vacancies will eventually be left? In what way will the remaining interstitials and vacancies form defects? (3) How does the intrinsic C vacancy affect the above two behaviors? Detailed analysis was carried out for the above three questions, as follows.

(1) The type, quantity and distribution of interstitials and vacancies generated by Au ion irradiation can be clearly decoded by SRIM software simulation. Irradiation produces Zr interstitials, C interstitials, Zr vacancies, and C vacancies. These point defects are presented in the form of Frenkel pairs (FP). Figure 1 shows the distribution of the Zr interstitials, C interstitials, Zr vacancies and C vacancies induced by ion irradiation along the irradiation direction. It can be found that the number of Zr interstitials and Zr vacancies generated by irradiation is slightly higher than the number of C interstitials and C vacancies, and both reach peaks at a depth of ~350 nm. The distribution of interstitials and vacancies is consistent with the distribution of dpa along the depth.

(2) How do the large number of Zr interstitials, C interstitials, Zr vacancies and C vacancies generated by irradiation move? There are two ways: one way is that the interstitials migrate to the atomic position, and the vacancies migrate to the vacancy position; the other way is that the interstitials recombine with the vacancies to form a combination of Frenkel pairs. 

The first type of migration is mainly determined by the migration barrier of interstitials and vacancies. Morgan et al. [22] used the first-principles method to calculate the migration barrier of interstitials and vacancies in ZrC, as shown in Table 3. The C vacancies and the Zr vacancies have very high migration barriers of 4.41 eV and 5.44 eV, respectively, which mean that they find it difficult to migrate at room temperature, meaning that the C vacancies and Zr vacancies generated by irradiation find it difficult to aggregate and grow. This therefore explains why no void is found in the cross-section TEM images of ZrC after irradiation. For C or Zr defects, interstitials have higher diffusivity than vacancies. The migration barrier of C interstitial is the lowest, at 0.27 eV; the migration barrier of Zr interstitial is 0.45 eV. Compared with vacancies, interstitials have higher diffusivity than vacancies, indicating that the defects formed after irradiation are mainly determined by interstitials.

In the second way, interstitials recombine with vacancies to form a recombination of Frenkel pairs, which is mainly determined by the recombination barrier of FP. The recombination barrier of Zr FP is about 0.32 eV [22], which is slightly lower than the migration barrier of the Zr interstitials (0.45 eV), indicating that most of the Zr interstitials tend to recombine with the Zr vacancies after irradiation. The recombination barrier of C FP in ZrC is as high as 1.66 eV, which is much higher than the migration barrier of C interstitials (0.27eV), indicating that most C interstitials find it difficult to recombine with C vacancies after irradiation and tend to migrate along a certain crystal plane to form defects such as dislocations/dislocation loops. Therefore, a large number of dislocations and dislocation loops were observed in the TEM photograph of ZrC after irradiation, indicating that the dislocation loops formed in ZrC belong to a carbon core interstitial-type loop.

(3) How does the intrinsic C vacancy affect the above two behaviors? From the SRIM simulation results (Figure 3), it is known that as the intrinsic C vacancies increase, the concentration of C interstitials generated by irradiation decreases. In addition, Morgan et al. [22] found that the intrinsic C vacancies in ZrC*_x_* can significantly reduce the recombination barrier of C FP. Therefore, compared with ZrC, C interstitials are more likely to recombine with C vacancies as the intrinsic C vacancies increase. In addition, the total C vacancy concentration increases due to the presence of the intrinsic C vacancies in ZrC*_x_*, increasing the probability of the C interstitials recombining with C vacancies. Therefore, for the non-stoichiometric ZrC*_x_*, the decrease in the number of C interstitials caused by irradiation, the low recombination barrier of C FP, and the high concentration of C vacancy can reduce the total number of C interstitials after cascade cooling, suppressing the formation and growth of the dislocation loop. Thus, the size of the dislocation loop of ZrC_0.9_ and ZrC_0.8_ in the TEM images after irradiation is smaller than that in ZrC. With the further increase in the intrinsic C vacancy concentration in ZrC*_x_*, the recombination barrier of C FP can be reduced to 0.2 eV [22], which is lower than the migration barrier of the C interstitial (0.27 eV). Therefore, most of the C interstitials after irradiation tend to recombine with C vacancies, making it difficult for them to form dislocation loops along the specific crystal plane. This explains why no dislocation loops caused by irradiation were observed in ZrC_0.7_ with higher intrinsic C vacancies.

As the intrinsic vacancies increased, the lattice expansion of ZrC*_x_* after irradiation decreases, and the formation and growth of dislocation loops is suppressed, which is significant for the enhancement of the tolerance of radiation damage, which yields non-stoichiometric ZrC*_x_* a promising candidate in the TRISO-coating fuel particle, which would be used to replace, or in addition to, the currently used SiC.

## 5. Conclusions

In the present work, ZrC*_x_* ceramics with different stoichiometries were irradiated with a four MeV Au ion beam at room temperature in doses of 2 × 10^16^ ions/cm^2^, corresponding to ~130 dpa. The ZrC*_x_* lattice was expanded after irradiation. With the decrease of the C/Zr ratio, the lattice expansion of ZrC*_x_* after irradiation decreases. The lattice swelling of ZrC_0.7_ was only 0.019%. The defect structures in ZrC, ZrC_0.9_ and ZrC_0.8_ are similar after irradiation. The NaCl type crystal structure of ZrC maintained after irradiation. Some long dislocation lines are formed at the near surface, while in the area with the greatest damage (depth of ~400 nm), large amounts of dislocation loops formed, and, as the C vacancy concentration increased, the size of the dislocation loops gradually decreased. Few dislocation loops were found in ZrC_0.7_ after irradiation, and only black-dot defects were found in the area with the greatest damage. For the non-stoichiometric ZrC*_x_*, as the intrinsic vacancies increased, the decrease in the number of C interstitials caused by irradiation, the low recombination barrier of C Frenkel pairs and the high concentration of C vacancies can reduce the total number of C interstitials after cascade cooling. Therefore, the formation and growth of dislocation loops was suppressed, which is significant for the enhancement of tolerance of radiation damage.

## Figures and Tables

**Table 1:** Basic properties of ZrC*_x_* with four stoichiometries and input parameters for SRIM simulation.

Materials		ZrC	ZrC_0.9_	ZrC_0.8_	ZrC_0.7_
Basic properties	Density (g/cm^3^)	6.59	6.49	6.41	6.31
	Relative density (%)	99.0	98.3	98.0	97.5
	Grain sizes (μm)	14.6 ± 5.8	13.5 ± 4.7	13.0 ± 4.4	13.5 ± 5.2
	Lattice parameters (Å)	4.6815 ± 0.0013	4.6810 ± 0.0031	4.6784 ± 0.0027	4.6776 ± 0.0011
Input parameters for the stopping and range of ions in matter version 2013 (SRIM-2013) simulation	Calculation type	Detailed Calculation with Full Damage Cascades			
	Ion species	Gold (Au)			
	Ion energy	Four MeV			
	Target width	1000 nm			
	Target density	6.59 g/cm^3^	6.49 g/cm^3^	6.41 g/cm^3^	6.31 g/cm^3^
	Target composition	ZrC	ZrC_0.9_	ZrC_0.8_	ZrC_0.7_
	Displacement energy	Zr = 37 eV, C = 16eV [22]			
	Number of ions	10000			

**Table 2:** Lattice parameters and lattice expansion of ZrC*_x_* with different stoichiometries before and after irradiation.

Materials		ZrC	ZrC_0.9_	ZrC_0.8_	ZrC_0.7_
Lattice parameters(Å)	Before irradiation	4.6815 ± 0.0013	4.6810 ± 0.0031	4.6784 ± 0.0027	4.6776 ± 0.0011
	After irradiation	4.6870 ± 0.0007	4.6848 ± 0.0025	4.6813 ± 0.0038	4.6785 ± 0.0024
Lattice expansion (%)		0.117	0.081	0.062	0.019

**Table 3:** Migration barriers of point defects following various diffusion mechanisms in ZrC [22].

Mechanism	Migration Path	Migration Barrier (eV)
C vacancy	Migration between nearest-neighbor sites on the C sublattice	4.41
Zr vacancy	Migration between nearest-neighbor sites on the Zr sublattice	5.44
C interstitial	Migration downward via a turning hop and diffuses to the right cell through a straight hop	0.27
Zr interstitialcy	Zr atom moves to the neighboring Zr site and kicks out the Zr atom	0.45
Zr interstitial	Zr atom diffuses from one interstitial site to another interstitial site	0.92
Zr kicks out C	Zr atom moves to the neighboring C site and kicks out a C atom	1.41
